# Special Issue “Mitochondrial Metabolism Alterations in Health and Disease”

**DOI:** 10.3390/ijms26104826

**Published:** 2025-05-18

**Authors:** Graziantonio Lauria, Rosita Curcio

**Affiliations:** Department of Pharmacy, Health and Nutritional Sciences, University of Calabria, Via P. Bucci, 87036 Rende, CS, Italy

Mitochondria are central hubs of cellular metabolism and signaling that play key roles in stress response, inflammation, calcium homeostasis, mitochondrial quality control, and cell death, with mitochondrial impairment potentially being the underlying cause of several conditions, including metabolic, neurodegenerative, and cardiovascular diseases. Furthermore, mitochondrial dysfunction can contribute to the hallmarks of cancer, including sustained proliferative signaling, resistance to cell death, and altered cellular energetics. Therefore, analyzing the mechanisms underlying mitochondrial dysfunction and developing targeted therapies to restore mitochondrial function have become essential research areas. This Special Issue offers an outstanding collection of up-to-date reviews and research articles focusing on mitochondria and their potential for fighting human diseases.

The article by Chandrasekarant et al. demonstrates that AMP-activated protein kinase (AMPK) activators could have therapeutic potential for diabetic peripheral neuropathy (DPN) by modulating mitochondrial metabolism [1]. The authors investigated the effects of the AMPK activator 5-Aminoimidazole-4-carboxamide 1-β-D-ribofuranoside (AICAR) in mouse models of Type 1 and Type 2 diabetes, finding that AICAR had preventive and reversing effects on experimental DPN. AICAR treatment promoted mitochondrial homeostasis through AMPK phosphorylation and activation. The latter resulted in increased levels of the mitochondrial fission marker dynamin-related protein 1, augmented phosphorylation of the autophagy-activating kinase, and increased levels of the autophagosome marker LC3-II, suggesting improved mitochondrial turnover and mitophagy. In mitochondria isolated from dorsal root ganglion neurons of mice that were fed high-fat diets, AICAR treatment enhanced mitochondrial function by increasing ADP-stimulated state 3 respiration. Furthermore, AICAR treatment improved systemic metabolic parameters, such as insulin sensitivity and glycemic and lipid profiles. All of these findings suggest that AMPK activators may act as exercise mimetics and neuroprotective agents, with therapeutic potential for the treatment of DPN, although further clinical studies in humans are still needed.

Levitt et al. demonstrated that short-term and chronic binge alcohol (CBA) non-synergistically decreased the differentiation capacity of myoblasts from simian immunodeficiency virus-infected female macaques [2]. The authors proved that short-term alcohol treatment decreased myoblast differentiation capacity and impaired cell bioenergetics by decreasing glycolytic function, ATP production, mitochondrial membrane potential and pyruvate kinase activity, while increasing the mitochondrial production of reactive oxygen species (ROS) and aldolase activity. Conversely the activity of the latter enzyme was found to be decreased, in chronically alcohol-fed rats. This suggests possible alternative metabolic fates of glucose in alcohol misuse. Potentially different mechanisms could be responsible for the decreased myoblast differentiation capacity following short-term alcohol consumption and CBA, highlighting the need to clarify the impact of different types of alcohol misuse on myopathy. These findings might be useful for attenuating the negative effects of alcohol abuse on mitochondrial bioenergetics, as well as for improving skeletal muscle mass and quality of life in subjects with alcohol use disorders.

Mitochondrial dysfunction has emerged as a significant pathogenetic factor in post-viral fatigue syndrome (PVFS), a condition that leads to low-grade systemic inflammation and includes myalgic encephalomyelitis, also known as chronic fatigue syndrome, fibromyalgia (FM), and post-COVID-19 (long COVID) illness. Decreased ATP production, immune dysregulation, and increased oxidative stress have been observed in PVFS patients, but no FDA-approved treatment is currently available. In their review, Mantle et al. discussed the efficacy of CoQ10 supplementation to improve mitochondrial function, reduce oxidative stress, and alleviate fatigue in PVFS patients [3]. The ability of CoQ10 to ameliorate mitochondrial dysfunction stems from the key role it plays in mitochondria. It is an electron carrier in the mitochondrial respiratory chain that can restore electron flow and enhance ATP generation. Moreover, CoQ10 is a fat-soluble antioxidant that protects mitochondria from damage caused by free radical species generated during oxidative phosphorylation. Some clinical trials showed promising results, especially in FM patients, where CoQ10 supplementation reduced pain and fatigue and improved energy generation.

Several authors critically reviewed the involvement of mitochondrial dysfunction in the pathogenesis of neurodegenerative disorders, including Parkinson’s disease (PD) and Alzheimer’s disease (AD). Neuronal functionality and survival require high energy levels to ensure the proper functioning of mitochondria. The article by Uspalenko et al. highlighted the involvement of mitochondrial ATP-dependent potassium channels in the development of PD. These authors demonstrated the protective effect of uridine in an experimental rat model of PD, induced by the injection of the neurotoxin, 6-hydroxydopamine (6-OHDA), into the substantia nigra [4]. 6-OHDA was used because it can inhibit complex I of the respiratory chain, mimicking the process that occurs in PD patients, where a reduction in the activity of this complex was observed. 6-OHDA inhibition increased ROS generation in dopaminergic neurons, thus causing severe mitochondrial dysfunction and impairing neuronal function, which resulted in motor deficit. Uridine treatment was able to increase the concentration of uridine diphosphate, a natural activator of these channels, resulting in reduced oxidative stress and mitochondrial dysfunction. Based on these findings, uridine treatment could be useful for preventing the development of PD and/or to treat this disease in its early stages.

Mitochondrial dysfunction plays a crucial role in mediating the intercorrelation between PD and associated cardiac comorbidities. Although the mechanisms underlying the increased prevalence of cardiac dysfunction observed in PD patients are still unclear, Salis Torres et al. reviewed some shared pathways capable of impairing mitochondrial function in both cardiac and neuronal tissues [5]. These authors highlighted numerous common pathways, including compromised mitochondrial bioenergetics; alterations in mitochondrial biogenesis and dynamics; the dysregulation of the PINK1/PRKN-mediated mitophagy pathway; disrupted mitochondrial calcium handling with decreased ATP production and increased oxidative stress; and excessive ROS production and lipid peroxidation resulting in cellular damage, protein aggregation, and mitochondrial dysfunction. These pathways are all common hallmarks of cardiac and neurological diseases, as well as genetic susceptibility, since certain PD-associated genes (PRKN, PINK1, and PARK7) are also expressed in cardiac tissue and play critical roles in mitochondrial quality control. This review provides insights into future research directions, as studying the role of mitochondrial dysfunction as a link between cardiac dysfunction and PD may offer an opportunity to reveal common metabolic pathways, leading to promising opportunities in research areas that aim to identify relevant shared therapeutic targets.

The comprehensive review by Yu et al. highlighted the genetic, proteomic, and metabolic alterations in AD linked to mitochondrial one-carbon metabolism [6]. The latter is involved in several processes, such as mitochondrial function, nucleotide synthesis, amino acid metabolism, and epigenetic regulation. Furthermore, it plays a central role in replenishing pools of nucleotides to preserve substrate bioavailability, which is useful for DNA repair. The authors proposed that multiple AD hallmarks, such as Aβ accumulation, tau hyperphosphorylation, and genetic risk factors converge on mitochondrial dysfunction. They suggested that enhancing mitochondrial one-carbon metabolism could help neurons counteract damage and delay AD-related pathologies, emphasizing the role of folate and NAD+ in mitigating mitochondrial dysfunction. The folate one-carbon pathway is critical for cellular health, as it supplies purine biosynthesis, takes part in the reduction of the essential redox cofactor NAD(P)+ to NAD(P)H, and allows the biosynthesis of the main methylation factor, S-adenosylmethionine. In addition, NAD+ and NADP+ are crucial cofactors in electron transfer and redox reactions. Defects in the function of respiratory chain complex I and low levels of NAD+ have been found in AD patients. Furthermore, NAD+ levels are also reduced in animal models of AD. On this basis, attempts have been made to restore cellular NAD+ levels in animal models of neurodegeneration with promising results. These approaches are currently undergoing clinical trials.

Other authors pointed out the critical involvement of mitochondria in cancer and how this could be exploited for new potential anti-cancer strategies, as well as for improving the diagnosis and prognosis of certain cancer types. 

The review by Moon provided an excellent overview of the role of ATP-sensitive potassium channels in cancer biology, highlighting their promising role as potential therapeutic targets in cancer treatment, as they modulate key cellular processes that are often dysregulated in cancer cells [7]. These channels are expressed in cell membranes, where they play a key role in linking cellular metabolism to electrical activity, as well as in mitochondria, where they modulate mitochondrial function, ATP generation, programmed cell death, and defense mechanisms against physiological stress. The structural features, regulation, mechanisms, expression, and mutations of these channels, as well as their role in modulating metabolic and ion balance, cell cycle progression, and proliferation in cancer cells are described in detail. Furthermore, the role of activators and inhibitors in cancer biology is extensively discussed. KATP channel activators, such as diazoxide, minoxidil, pinacidil, and cromakalim, can affect proliferation, invasion, drug delivery, and apoptosis in a context-dependent manner. In contrast, KATP channel inhibitors, particularly sulfonylurea drugs, such as glyburide, glipizide, and repaglinide, have shown promising anti-cancer properties, modulating signaling pathways involved in angiogenesis, cell cycle regulation, and cell death. According to the author, selectively disrupting the proliferation and survival of cancer cells requires a thorough understanding of the several roles that these channels play in different cancer cells, as their function can vary significantly depending on the environment and cancer cell type. Furthermore, a future challenge will be the design of potential drugs able to act specifically on mitochondrial channels without affecting those located in the plasma membrane.

The article by Frattaruolo et al. demonstrated the promising in vitro antitumor activity of some plant-derived flavanones in breast cancer cell lines [8]. The authors compared the selective and dose-dependent cytotoxic activity of three natural flavanones isolated from the leaves of Glycyrrhiza glabra—Glabranin, Pinocembrin, and Licoflavanone—in the breast cancer cell lines, MCF-7, MDA-MB-231, and MCF-10A. Licoflavanone exhibited the best cytotoxic and proapoptotic activity, along with the greatest ability to inhibit cell motility and reduce macrophage activation induced by breast-cancer-conditioned media. Furthermore, Licoflavanone treatment modulated breast cancer cell energy metabolism by decreasing the mitochondrial oxygen consumption rate linked to ATP production. Although all three tested compounds were able to inhibit the propagation and survival of cancer stem cells (CSCs) and reduce the expression of stemness markers in CSCs, Licoflavanone emerged as the most potent inhibitor. Notably, CSCs are a subpopulation of tumor cells with high metabolic flexibility, whose proliferation is largely dependent on mitochondrial function. These features increase their chemoresistance and ability to metastasize and relapse, and are often responsible for the failure of conventional therapeutic strategies. In this context, the study of molecules that are able to modulate mitochondrial bioenergetics could prove to be an opportunity for the development of basic research findings into possible future clinical applications, especially for those tumors that strictly depend on mitochondrial oxidative metabolism.

Nowadays, the diagnosis of thyroid cancer is mainly based on imaging techniques and cytological analysis. When the diagnosis is ambiguous, the quantification of molecular biomarkers is evaluated after cytological examination. Currently commercially available molecular tests are expensive and widely vary. Therefore, new molecular biomarkers with higher diagnostic reliability are being developed, as well as better classification of prognosis and recurrence. Current research has focused on different promising biomarkers for thyroid cancer. In this regard, the review by Cabané et al. provided a comprehensive overview of the current state of molecular markers for thyroid cancer diagnosis and prognosis, with a focus on emerging opportunities from non-coding RNAs and mitochondrial signatures [9]. Promising biomarkers related to non-coding genome include miRNAs, long non-coding RNAs, circular and double-stranded RNAs, while those related to mitochondria include mitochondrial DNA signatures, circulating cell-free mtDNA, and mitophagic gene signatures (expression of the mitophagic proteins FAM162A, NIX, and BNIP3). It is known that high mtDNA heteroplasmy results in increased ROS levels, which impairs mitochondrial bioenergetics and alters mitophagy, thus favoring the development of pathologies such as cancer. When mitochondrial stress and/or defective mitophagy occurs, mtDNA can escape from mitochondria and cells into the bloodstream, where it can be detected and measured. Therefore, the amount and features of circulating cell-free mtDNA represent useful parameters of mitochondrial dysfunction and disease. 

Overall, this Special Issue of *IJMS* provides a collection of nine articles that discuss various aspects of mitochondrial involvement in human diseases, aiming to find new promising therapeutic strategies to treat, prevent, and/or diagnose them. Different authors pointed out that some treatments that are able to restore mitochondrial function were effective in counteracting DPN, PVFS, and altered myoblast differentiation following alcohol misuse. A group of articles shed light on the critical role of mitochondria and associated pathways in neurodegenerative diseases, focusing on the latest knowledge and emphasizing the importance of understanding and harnessing mitochondrial pathways to counteract mitochondrial dysfunction, which underlies many of the defects found in these diseases. Possible approaches should target mitochondrial dynamics and bioenergetics to alleviate symptoms and progression. Another group of articles helped pave the way for new research and possible therapeutic approaches in the fight against cancer, focusing on modulating mitochondrial function. In this context, one of the most important future challenges of personalized medicine will be the development of strategies capable of selectively targeting tumor cells while minimizing damage to healthy cells. In addition, the promising role of emerging biomarkers for improving the diagnosis and prognosis of thyroid cancer is discussed. All of this knowledge can provide new and valuable insights for future research directions.
